# Association of total bilirubin and indirect bilirubin content with metabolic syndrome among Kazakhs in Xinjiang

**DOI:** 10.1186/s12902-020-00563-y

**Published:** 2020-07-22

**Authors:** Hao Hao, Heng Guo, Ru-lin Ma, Yi-zhong Yan, Yun-hua Hu, Jiao-long Ma, Xiang-hui Zhang, Xin-ping Wang, Kui Wang, La-ti Mu, Yan-peng Song, Jing-yu Zhang, Jia He, Shu-xia Guo

**Affiliations:** 1 Department of Public Health, Shihezi University School of Medicine, Shihezi, 832000 Xinjiang China; 2 The First Affiliated Hospital of Shihezi University Medical College, Shihezi, 832000 Xinjiang China; 3 Department of Pathology and Key Laboratory of Xinjiang Endemic and Ethnic Diseases (Ministry of Education), Shihezi University School of Medicine, Shihezi, 832000 Xinjiang China

**Keywords:** Bilirubin, Transaminase, Metabolic syndrome

## Abstract

**Background:**

Some studies have shown that a high level of bilirubin is a protective factor against metabolic syndrome (MS), while a high level of transaminase is a risk factor for MS. However, the existing results are inconsistent and few cohort studies have been published.

**Methods:**

Using an ambispective cohort study, 565 Kazakhs from Xinjiang, China were selected as the study subjects. The baseline serum bilirubin and transaminase levels of the subjects were divided into quartiles and the relationship between these values and the incidence of MS was analyzed. The definition of MS was based on the Joint Interim Statement (JIS) diagnostic criteria.

**Results:**

The average follow-up time for the subjects was 5.72 years. The cumulative incidence of MS was 36.11% (204 of the 565 subjects), and the incidence density was 63.10/1000 person-years. Multivariate Cox regression analysis showed that the levels of total bilirubin (TBIL) and indirect bilirubin (IBIL) were negatively correlated with the occurrence of MS, Compared to the lowest quartile level (Q1), the hazard ratios of MS the TBIL levels at the Q2-Q4 quartiles were: 0.47 (0.31–0.71), 0.53 (0.35–0.79), and 0.48 (0.32–0.72), respectively, while IBIL levels at the Q2-Q4 quartiles showed an MS hazard ratio of 0.48 (0.32–0.72), 0.54(0.36–0.81), and 0.52 (0.35–0.77), respectively, all at a 95% confidence level. However, no relationship was found between transaminase levels and the incidence of MS.

**Conclusion:**

Serum TBIL and IBIL levels were negatively correlated with the incidence of MS in a Kazakh population in China.

## Background

Metabolic syndrome (MS) is a clustering of complex medical conditions, including hypertension, hyperglycemia, central obesity, hypertriglyceridemia, and high-density lipoprotein cholesterol (HDL-C) reduction. MS can lead to cardiovascular disease, type 2 diabetes, lipid disorder, liver steatosis, and other circulatory system disorders [1]. Although the etiology and pathogenesis of MS are not fully understood, it is generally believed that factors such as insulin resistance, inflammation, and oxidative stress play an important role in its occurrence and development [1–3]. Bilirubin, the main metabolite of heme, is toxic, and can cause irreversible damage to the brain and nervous system. Liver cells remove bilirubin from the body through its intake, transformation, and excretion. High levels of bilirubin in the serum indicate liver dysfunction. However, increasing evidence suggests that serum bilirubin has anti-inflammatory and antioxidant functions, and can also improve vascular endothelial function and enhance insulin sensitivity [4, 5], suggesting that a high level of bilirubin can protect against MS [6, 7]. Transaminase can promote inflammation and induce insulin resistance [8] and studies have shown that a high level of the transaminase alanine aminotransferase (ALT) is a risk factor for MS [6, 9]. One cohort study showed that γ-glutamyl transpeptidase levels in men and ALT levels in women are related to MS, but that direct bilirubin (DBIL), total bilirubin (TBIL), ALT, and aspartate aminotransferase (AST) levels in men and DBIL, TBIL, and AST levels in women are not [10]. However, further research is clearly needed on this topic, as the results of the previous studies are inconsistent.

Kazakhs in Xinjiang, China are nomadic minorities living primarily in remote mountainous areas. The majority fall into a low economic class and their culture and customs are significantly different from those of other ethnic groups [11, 12]. A previous study by our research group found the prevalence of MS in Kazakhs to be higher than that in other ethnic groups in Xinjiang and higher than the national average [13]. Therefore, this study used Xinjiang Kazakh residents as the research group and explored the relationship between the incidence of MS and serum bilirubin and transaminase levels. The results of this study provide potential advances for the early diagnosis and prevention of MS in the Xinjiang Kazakh population; our results may be extended to people living in Kazakhstan and Uzbekistan as well.

## Methods

### Research subjects

Based on the geographical distribution of Xinjiang’s ethnic minorities in northwestern China, a representative county (six villages in the Nalati Township, Xinyuan County) was selected and an ambispective cohort study was conducted on the local Kazakh population. The study baseline was conducted in 2009 and 2012 and a follow-up was performed in 2013, 2016, and 2017. The study included 785 Kazakh permanent residents aged 18 and over who voluntarily participated in the survey; 10 subjects with chronic liver disease, 2 subjects with cancer, and 208 subjects with MS at baseline were excluded from the study, resulting in a total of 565 subjects.

### Epidemiological investigation and biochemical tests

At baseline and at each follow-up, a face-to-face interview was conducted by uniformly trained investigators to collect personal information, disease history, family history, and lifestyle behavior. Height, weight, waist circumference (WC), systolic blood pressure (SBP), and diastolic blood pressure (DBP) were measured and recorded according to standardized methods. TBIL, DBIL, indirect bilirubin (IBIL), ALT, AST, fasting plasma glucose (FPG), triglyceride (TG), total cholesterol (TC), HDL-C, and low-density lipoprotein cholesterol (LDL-C) levels were measured using an automatic biochemical analyzer (Olympus Au 2700; Olympus diagnostics, Hamburg, Germany). Each subject signed an informed consent form. The investigation was approved by the ethics review committee of the First Affiliated Hospital of Shihezi University Medical College (IERB No. SHZ2010LL01), and the study methods were carried out in accordance with the relevant guidelines.

### Definition of MS

Our identification of MS is based on the Joint Interim Statement (JIS) diagnostic criteria [14]. For MS to be diagnosed, three or more of the following indicators must be observed: (1) men WC ≥ 85 cm, women WC ≥ 80 cm; (2) SBP ≥ 130 mmHg or DBP ≥ 85 mmHg, have received treatment for hypertension, or have been previously diagnosed with hypertension; (3) FPG ≥ 5.6 mmol/L, have received treatment for type 2 diabetes, or have been previously diagnosed with type 2 diabetes. (4) TG ≥ 1.70 mmol/L or received treatment for elevated triglycerides; (5) men HDL-C < 1.0 mmol/L, women HDL-C < 1.30 mmol/L.

### Statistical analysis

Data analysis was performed using SPSS version 20.0 (Chicago, Illinois, USA). Means ± standard deviations were used to describe continuous variables, and percentages were used to describe categorical variables. Rank sum tests were used to compare baseline characteristics between men and women, and chi-square tests were used to compare categorical variables. Serum bilirubin and transaminase levels in this population differed between men and women. Therefore, the baseline levels were separated into quartiles (Q1, Q2, Q3, and Q4) for each gender. The multivariate Cox proportional-hazards model was used to analyze the relationship between serum bilirubin or transaminase levels and MS. After adjusting for age and gender, the hazard ratio (HR) and 95% confidence interval (95% CI) of serum bilirubin and transaminase levels to MS were calculated. All statistical tests were bilateral and *P* < 0.05 indicated statistical significance.

## Results

### Baseline characteristics

A total of 565 subjects were included in this survey, including 221 males (39.1%). The average age of the population was 40.33 ± 11.86 years; the average age of men was significantly higher than that of women (men = 42.10 ± 12.93 years; women = 39.20 ± 10.99 years; *P* = 0.011). In a comparison of baseline characteristics between the two genders, the levels of WC, SBP, DBP, TG, TBIL, IBIL, ALT, and AST were higher in men than women, and HDL-C levels were higher in women than in men. These differences were statistically significant (*P* < 0.05). These data are shown in Table 1.

**Table 1:** Baseline characteristics based on different genders

	Total	Men	Women	*P*
Number (%)	565	221 (39.1)	344 (60.9)	
Age (years)	40.33 ± 11.86	42.10 ± 12.93	39.20 ± 10.99	**0.011**
Smoking, N (%)	187 (33.1)	117 (52.9)	70 (20.3)	**< 0.001**
Drinking, N (%)	55 (9.7)	52 (23.5)	3 (0.9)	**< 0.001**
WC (cm)	81.58 ± 9.97	83.64 ± 9.77	80.26 ± 9.88	**< 0.001**
SBP (mmHg)	127.84 ± 21.72	132.10 ± 21.64	125.11 ± 21.36	**< 0.001**
DBP (mmHg)	81.92 ± 13.79	84.06 ± 13.52	80.54 ± 13.81	**< 0.001**
FPG (mmol/L)	4.37 ± 0.97	4.48 ± 1.12	4.29 ± 0.86	0.214
TG (mmol/L)	1.05 ± 0.84	1.05 ± 0.52	1.05 ± 0.99	**0.018**
TC (mmol/L)	4.13 ± 0.94	4.17 ± 0.86	4.11 ± 0.99	0.212
HDL-C (mmol/L)	1.42 ± 0.37	1.36 ± 0.36	1.46 ± 0.38	**< 0.001**
LDL-C (mmol/L)	2.08 ± 0.67	2.13 ± 0.64	2.04 ± 0.69	0.054
BMI (kg/m^2^)	23.26 ± 3.44	23.43 ± 3.32	23.15 ± 3.51	0.359
TBIL (μmol/L)	10.84 ± 4.70	11.84 ± 4.71	10.19 ± 4.59	**< 0.001**
IBIL (μmol/L)	8.08 ± 3.82	8.76 ± 3.88	7.65 ± 3.73	**< 0.001**
DBIL (μmol/L)	2.58 ± 1.48	2.72 ± 1.56	2.48 ± 1.42	0.054
ALT (IU/L)	15.34 ± 10.36	16.43 ± 8.28	14.64 ± 11.45	**< 0.001**
AST (IU/L)	27.37 ± 12.48	28.70 ± 11.84	26.52 ± 12.82	**0.002**

*WC* waist circumference; *SBP* systolic blood pressure; *DBP* diastolic blood pressure; *FPG* fasting plasma glucose; *TG* Triglycerides; *TC* total cholesterol, *HDL-C* high-density lipoprotein cholesterol, *LDL-C* low-density lipoprotein cholesterol; *BMI* body mass index; *TBIL* total bilirubin; *IBIL* indirect bilirubin; *DBIL* direct bilirubin, *ALT* alanine aminotransferase; *AST* aspartate aminotransferase

### Incidence of MS

Follow-up was conducted with the subjects for 3233.04 person-years, with an average follow up time of 5.72 ± 1.49 years per subject. During this period, a total of 204 subjects developed MS, with a cumulative incidence of 36.11% (95% CI = 0.32–0.40) and an incidence density of 63.10/1000 person-years. Of the 221 men in the study, 70 developed MS, with a cumulative incidence of 31.67% (95% CI = 0.26–0.38) and an incidence density of 55.71/1000 person-years. The cumulative incidence increased with age (χ^2^ trend = 43.166, *P* < 0.001). Of the 344 women in this study, 134 developed MS, with a cumulative incidence of 38.95% (95% CI = 0.34–0.44) and an incidence density of 67.80/1000 person-years. The cumulative incidence again increased with age (χ^2^ trend = 76.391, *P* < 0.001). There was no statistically significant difference in the cumulative incidence and incidence density between men and women. These data are shown in Table 2.

**Table 2:** Comparison of the incidence of metabolic syndrome in different age groups

Group	Number	Number of MS	Cumulative incidence (%)	Incidence density (10^−3^person-years)	χ^2^_trend_	P
total	565	204	36.11	63.10		
men	221	70	31.67	55.71		
< 35	69	14	20.29	36.75	43.166	**< 0.001**
35–45	70	23	32.86	54.96		
> 45	82	33	40.24	72.19		
women	344	134	38.95	67.80		
< 35	128	38	29.69	51.49	76.391	**< 0.001**
35–45	121	51	42.15	72.24		
> 45	95	45	47.37	84.52		

### Analysis of factors influencing MS

We divided the levels of serum bilirubin and transaminase into quartiles and examined the relationship between the different quartile levels and the risk of MS. There was no significant difference in the cumulative incidence of MS at different quartile levels. Using serum bilirubin and transaminase levels as independent variables and the occurrence of MS as the dependent variable for Cox proportional hazards model analysis and adjusting for age and gender, we found that TBIL and IBIL levels were negatively correlated with MS, but there was no correlation between the DBIL, ALT, or AST levels and MS (Table 3). Compared to the lowest quartile level (Q1), the HRs (95% CI) of TBIL Q2-Q4 levels were 0.47 (0.31–0.71), 0.53 (0.35–0.79), and 0.48 (0.32–0.72), respectively, and those of the IBIL Q2-Q4 levels were 0.48 (0.32–0.72), 0.54(0.36–0.81), and 0.52 (0.35–0.77), respectively. However, as shown in Table 3, there was no correlation between the DBIL, ALT, or AST levels and MS.

**Table 3:** Analysis of influencing factors for incident metabolic syndrome

Parameter	n	MS,n(%)	*HR*	*95%CI*	*P*
TBIL					
Q1	139	46 (33.1)	ref	ref	ref
Q2	141	50 (35.5)	**0.47**	**0.31–0.71**	**< 0.001**
Q3	143	53 (37.1)	**0.53**	**0.35–0.79**	**0.002**
Q4	142	55 (38.7)	**0.48**	**0.32–0.72**	**< 0.001**
IBIL					
Q1	140	46 (32.9)	ref	ref	ref
Q2	141	48 (34.0)	**0.48**	**0.32–0.72**	**< 0.001**
Q3	139	53 (38.1)	**0.54**	**0.36–0.81**	**0.003**
Q4	145	57 (39.3)	**0.52**	**0.35–0.77**	**0.001**
DBIL					
Q1	133	43 (32.3)	ref	ref	ref
Q2	135	54 (40.0)	1.23	0.82–1.84	0.320
Q3	150	56 (37.3)	1.32	0.88–1.96	0.178
Q4	147	51 (34.7)	0.88	0.58–1.32	0.531
ALT					
Q1	115	33 (28.7)	ref	ref	ref
Q2	194	68 (35.1)	0.99	0.65–1.50	0.949
Q3	131	55 (42.0)	1.06	0.69–1.63	0.806
Q4	125	48 (38.4)	1.04	0.67–1.62	0.857
AST					
Q1	130	44 (33.8)	ref	ref	ref
Q2	159	69 (43.4)	0.76	0.52–1.12	0.169
Q3	136	49 (36.0)	0.70	0.47–1.06	0.091
Q4	140	42 (30.0)	0.86	0.56–1.31	0.475

Analysis adjusted for age, sex

### Correlation analysis of serum bilirubin and transaminase levels and MS at baseline with different components

In the populations with zero or one MS component at baseline, multivariate Cox regression analysis showed that the parameters shown in Table 4 are not related to the risk of MS. In the population with two MS components at baseline, TBIL and IBIL levels were negatively correlated with the risk of MS. Compared to the lowest quartile level (Q1), the risk HRs (95% CI) of the TBIL Q2-Q4 group were 0.35 (0.21, 0.60), 0.36 (0.21, 0.62), and 0.38 (0.22, 0.64), respectively, and those of the IBIL Q2-Q4 group were 0.31 (0.18–0.54), 0.39 (0.23–0.66), and 0.31 (0.18–0.54), respectively. However, DBIL, ALT, and AST levels were not related to the risk of MS (Table 4).

**Table 4:** Analysis of serum bilirubin, transaminase and MS at baseline with different components

	MS component number = 0 (*n* = 104)		MS component number = 1 (*n* = 237)		MS component number = 2 (*n* = 224)	
	*HR(95%CI)*	*P*	*HR(95%CI)*	*P*	*HR(95%CI)*	*P*
TBIL						
Q1	ref	ref	ref	ref	ref	ref
Q2	3.80 (0.44–33.02)	0.226	0.66 (0.31–1.42)	0.284	**0.35 (0.21–0.60)**	**< 0.001**
Q3	5.00 (0.62–40.15)	0.130	0.73 (0.35–1.56)	0.419	**0.36 (0.21–0.62)**	**< 0.001**
Q4	3.53 (0.44–28.74)	0.238	0.67 (0.31–1.43)	0.299	**0.38 (0.22–0.64)**	**< 0.001**
IBIL						
Q1	ref	ref	ref	ref	ref	ref
Q2	3.14 (0.35–28.55)	0.309	0.57 (0.28–1.17)	0.123	**0.31 (0.18–0.54)**	**< 0.001**
Q3	6.81 (0.85–54.62)	0.071	0.41 (0.19–0.87)	0.020	**0.39 (0.23–0.66)**	**< 0.001**
Q4	3.69 (0.46–29.87)	0.221	0.74 (0.39–1.41)	0.355	**0.31 (0.18–0.54)**	**< 0.001**
DBIL						
Q1	ref	ref	ref	ref	ref	ref
Q2	0.77 (0.20–3.03)	0.707	1.29 (0.63–2.64)	0.490	1.36 (0.80–2.31)	0.254
Q3	1.66 (0.54–5.11)	0.376	1.27 (0.59–2.70)	0.541	1.30 (0.76–2.22)	0.333
Q4	0.31 (0.07–1.39)	0.125	1.19 (0.60–2.39)	0.618	1.03 (0.59–1.80)	0.919
ALT						
Q1	ref	ref	ref	ref	ref	ref
Q2	0.53 (0.16–1.76)	0.301	0.88 (0.44–1.73)	0.703	1.23 (0.68–2.23)	0.502
Q3	0.95 (0.31–2.92)	0.932	1.02 (0.51–2.06)	0.947	1.05 (0.58–1.98)	0.877
Q4	0.47 (0.13–1.80)	0.273	0.79 (0.35–1.77)	0.564	1.24 (0.67–2.28)	0.496
AST						
Q1	ref	ref	ref	ref	ref	ref
Q2	1.71 (0.53–5.55)	0.371	0.84 (0.42–1.68)	0.625	0.58 (0.35–0.97)	0.038
Q3	1.59 (0.44–5.73)	0.477	0.57 (0.28–1.16)	0.119	0.67 (0.39–1.17)	0.160
Q4	0.88 (0.19–4.08)	0.873	0.75 (0.36–1.59)	0.454	0.82 (0.47–1.44)	0.498

The model with adjustment for age, sex

## Discussion

In the Xinjiang Kazakh population, we determined the incidence of MS according to the JIS diagnostic criteria and analyzed the association of serum bilirubin and transaminase levels with MS. To our knowledge, the above study may be the first study for this specific population. The cumulative incidence of MS was 36.11% (95% CI = 0.32,0.40) and the incidence density was 63.10/1000 person-years. Recent studies have shown that the cumulative incidence of MS in China in 7 years was 18.55% [15] and the incidence density of MS in rural areas of South Korea was 30/1000 person-years for men and 46.4/1000 person-years for women [16]. The cumulative incidence and incidence density of MS in the Xinjiang Kazakh population are significantly higher than those in the groups mentioned above. This may be related to the unique genetic background, lifestyle, and eating habits of the Kazakh people. Staple foods of the Kazakh diet include roasted naan with flour and salt, cured meat (beef and mutton), and salty milk tea. In addition, women, especially, elderly women after marriage, rarely participate in work, field labor, and outdoor activities. The above factors lead to a higher incidence of hypertension, obesity, and dyslipidemia, which can explain the higher incidence of MS in the Kazakh population.

This study found that as the levels of TBIL and IBIL increase, the risk of MS is gradually reduced, suggesting that high levels of bilirubin have a protective effect against MS. Bilirubin is the main metabolite of heme and has long been considered a marker of liver dysfunction. Previous studies have shown that low levels of serum bilirubin increase the risk of cardiovascular diseases, diabetes, MS, obesity, and other diseases, while mildly increased serum bilirubin levels confer protection against these diseases [17]. Gilbert syndrome is a liver disorder characterized by benign hyperbilirubinemia, and studies have shown that Gilbert syndrome offers cardiovascular protection [18]. Studies have also shown that individuals with elevated bilirubin levels have a reduced risk of cardiovascular disease occurrence and death [19]. Bilirubin is recognized as the most effective endogenous antioxidant due to its continuous production in the bilirubin/biliverdin redox cycle, which can effectively scavenge hydrogen peroxide free radicals and inhibit lipid peroxidation of serum LDL [20, 21]. Bilirubin is 20 times more effective than the vitamin E analogue Trolox in preventing LDL oxidation [22]. Other studies have found that bilirubin has an anti-inflammatory effect. C-reactive protein (CRP) is a marker of chronic inflammation; several studies have shown that serum bilirubin levels are negatively correlated with CRP levels [23, 24]. Bilirubin can also inhibit the over-expression of vascular adhesion molecules induced by the inflammatory cytokine TNF-α [25]. In addition, mildly elevated bilirubin levels may increase insulin sensitivity. In experimental studies, bilirubin can improve the insulin sensitivity in leptin receptor-deficient and diet-induced mice with obesity [26, 27].

In view of the effects of bilirubin, some other studies have found a negative correlation between the total serum bilirubin levels and MS, which is consistent with the results of our study [7, 28, 29]. The results of some other studies, however, are not consistent with those of the current study. No correlation between baseline TBIL levels and MS was observed in Japanese men and women [30], and the relationship between the bilirubin subtype and MS was also inconsistent. A five-year cohort study in Chinese men showed that the levels of DBIL, but not those of TBIL and IBIL, were negatively correlated with MS [31]. This directly contradicts the results of the current study, which showed that serum levels of TBIL and IBIL, but not DBIL, were negatively correlated with MS. However, many studies have found that IBIL (also known as unconjugated bilirubin, UCB) has anti-inflammatory and anti-oxidant effects. In patients with Gilbert syndrome (increased serum UCB levels), serum bilirubin concentration was inversely related to the levels of oxidative stress markers [32]. Clinical research shows that elevated UCB levels can reduce the expression of pro-inflammatory cytokines and increase the body’s antioxidant capacity [33]. In addition, evidence from experimental studies in vitro and in vivo strongly supports the data obtained in human studies. A study on the treatment of experimental colitis in a rat model showed that UCB treatment reduced the inflammatory response induced by trinitrobenzene sulfonic acid and had a strong anti-inflammatory effect [34]. Hypochlorous acid (HOCl), an oxidant produced by myeloperoxidase (MPO), can induce protein and lipid oxidation. An in vitro experimental study showed that the addition of exogenous UCB to the serum and plasma of humans and rats can protect proteins and lipids from MPO-induced oxidation and reduce the production of chloramine and its decomposition products induced by HOCl and MPO [35].

Our study on the Kazakh population of Xinjiang did not show a relationship between transaminase levels and the risk of MS. This is inconsistent with the results of other studies [6, 9]. There are two possible explanations for this: (1) the sample size of the study population is small and/or the test efficiency is low; and (2) the genetics, diets, and living habits of the study population in our study are too different from those in the previous studies.

Our study found a negative correlation between TBIL or IBIL levels and the risk of MS in a study population with two MS components at baseline. In the study population with zero or one MS component at baseline, the levels of TBIL, IBIL, DBIL, ALT, and AST were not correlated with risk of MS. This may be due to the fact that when the research subject has two components of MS, the metabolic disorder is more severe. The serum bilirubin can clear superoxide and peroxidative free radicals produced due to MS, inhibit lipid peroxidation, and fight oxidative stress, thereby enhancing the protective effects, such as reduced inflammatory response.

There are some limitations of our research. First, the small sample size of this study may limit the validity of our findings. Second, we did not measure parameters of oxidative stress, inflammatory mediators, and bilirubin metabolism-related enzymes in the study subjects. Third, each subject’s bilirubin and transaminase levels were measured only once at each physical examination, which would not account for possible short-term fluctuations. Fourth, the present study did not collect information on comorbidities and medication use for data analysis.

## Conclusions

This study found that serum TBIL and IBIL levels were negatively correlated with MS in the Kazakh population in Xinjiang, China; these findings may provide new options for the treatment and prevention of MS. Certain medications can be used to increase serum bilirubin levels, but there are safety concerns that will need to be addressed via further studies. Serum bilirubin levels may also be increased through nonpharmacological means, such as quitting smoking, weight loss, and the intake of the food supplement phycobilin [36, 37].

## Data Availability

The datasets generated during and/or analysed during the current study are not publicly available due the papers written using this dataset have not been published but are available from the corresponding author on reasonable request.

## Abbreviations

MS: Metabolic syndrome
JIS: Joint Interim Statement
TBIL: Total bilirubin
IBIL: Indirect bilirubin
DBIL: Direct bilirubin
ALT: Alanine aminotransferase
AST: Aspartate aminotransferase
WC: Waist circumference
SBP: Systolic blood pressure
DBP: Diastolic blood pressure
FPG: Fasting plasma glucose
TG: Triglyceride
TC: Total cholesterol
HDL-C: High-density lipoprotein cholesterol
LDL-C: Low-density lipoprotein cholesterol
HR: Hazard ratio
95% CI: 95% confidence interval
CRP: C-reactive protein
UCB: Unconjugated bilirubin
HOCl: Hypochlorous acid
MPO: Myeloperoxidase
